# Precursor-Directed Generation of Indolocarbazoles with Topoisomerase IIα Inhibitory Activity

**DOI:** 10.3390/md16050168

**Published:** 2018-05-17

**Authors:** Cong Wang, Adeep Monger, Liping Wang, Peng Fu, Pawinee Piyachaturawat, Arthit Chairoungdua, Weiming Zhu

**Affiliations:** 1 Key Laboratory of Marine Drugs, Ministry of Education of China, School of Medicine and Pharmacy, Ocean University of China, Qingdao 266003, China; wangcong123206@163.com (C.W.); fupeng@ouc.edu.cn (P.F.); 2 Guangxi Key Laboratory of Chemistry and Engineering of Forest Products, School of Chemistry and Chemical Engineering, Guangxi University for Nationalities, Nanning 530006, China; 3 Toxicology Graduate Program, Department of Physiology, Faculty of Science, Mahidol University, Bangkok 10400, Thailand; adeepmonger11@gmail.com (A.M.); pawinee.pia@mahidol.ac.th (P.P.); 4 Key Laboratory of Chemistry for Natural Products of Guizhou Province and Chinese Academy of Sciences, Guiyang 550002, China; lipingw2006@163.com; 5 Toxicology Graduate Program, Excellent Center for Drug Discovery (ECDD), Faculty of Science, Mahidol University, Bangkok 10400, Thailand; 6 Laboratory for Marine Drugs and Bioproducts, Qingdao National Laboratory for Marine Science and Technology, Qingdao 266003, China

**Keywords:** marine-derived Streptomyces, *Streptomyces* sp. OUCMDZ-3118, indolocarbazole, cytotoxicities, topoisomerase IIα enzyme activity

## Abstract

One new indolocarbazole, 3-hydroxy-K252d (**3**), together with the recently reported 3-hydroxyholyrine A (**1**) and 3′-*N*-acetyl-3-hydroxyholyrine A (**2**), were obtained by feeding a culture of the marine-derived *Streptomyces* strain OUCMDZ-3118 with 5-hydroxy-l-tryptophan. Their structures were elucidated on the basis of spectroscopic analysis. Compound **1** potently induced apoptosis of gastric cancer cells by inhibiting topoisomerase IIα enzyme activity and reducing the expression of antiapoptosis protein level. Compound **3** displayed moderate cytotoxicity against the A549 and MCF-7 cell lines with IC_50_ values of 1.2 ± 0.05 μM, 1.6 ± 0.09 μM, respectively.

## 1. Introduction

Indolocarbazoles (ICZs) are a group of naturally occurring compounds with an indolo[2,3-*a*]pyrrolo[3,4-*c*]carbazole skeleton, which have been identified from different natural sources including fungi, actinomyces, and invertebrates [1]. It is an attractive class of molecules due to their powerful biological activities and interesting chemical structures [1,2]. To date, more than 140 ICZ alkaloids have been identified [1]. Among them, staurosporine, the first ICZ isolated from a Streptomyces strain in 1977 [3], is the most outstanding compound for its potent protein kinase C inhibition, although it has not been used as an antitumor drug because of its high toxicity and low selectivity. However, profound research on staurosporine has made an impact on future drug development of other ICZs. For example, ICZ analogues, PKC-412, and CEP-701 have been approved by the Food and Drug Administration (FDA) as a drug and orphan drug for the treatment of acute myeloid leukemia with or without Flt3-ITD mutation, respectively [4,5,6,7,8]. Our previous works reported several new bioactive ICZs from marine-derived actinobacteria, such as ZHD-0501 [9], fradcarbazoles A–C [10], and streptocarbazoles A and B [11].

**Figure 1:**
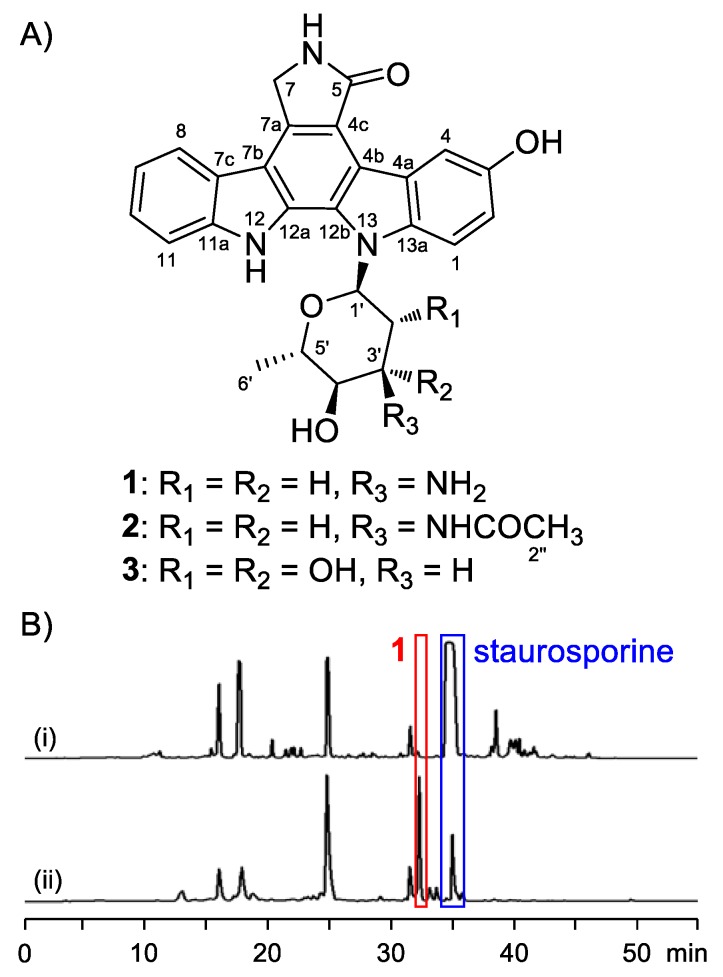
Precursor-directed generation of compounds **1**–**3**. (**A**) Structures of **1**–**3**. (**B**) HPLC analysis of *Streptomyces* sp. OUCMDZ-3118 cultures grown in the absence (i) or presence (ii) of 5-hydroxy-l-tryptophan.

While ICZs have been utilized as the starting core of many chemical syntheses and biological studies [1,12,13,14], there remains considerable scope for obtaining more active analogues through a simple route: precursor-directed biosynthesis [15,16,17]. We have modified the carbazole ring by feeding a culture of *Streptomyces* sp. OUCMDZ-3118 with 5-hydroxy-l-tryptophan to obtain new ICZ with the hydroxy substitution on the indole nucleus. Chemical investigation on those feeding experiments resulted in the identification of one new hydroxy substituted ICZ, 3-hydroxy-K252d (**3**) (Figure 1), two recently reported indolocarbazoles, 3-hydroxyholyrine A (**1**), and 3′-*N*-acetyl-3- hydroxyholyrine A (**2**) [18]. Compound **1** exhibited potent cytotoxicity against two gastric cancer cell lines, AGS and MKN45. This compound potently inhibited topoisomerase IIα activity, leading to DNA damage, and thereby inducing apoptotic cell death. In addition, compound **1** also reduced the expression of an antiapoptotic protein survivin that is involved in tumorigenesis.

## 2. Results and Discussion

### 2.1. Structure Elucidation

Compounds **1** and **2** were identified as the recently published 3-hydroxyholyrine A and 3′-*N*-acetyl-3-hydroxyholyrine A by specific rotation, high-resolution electrospray mass ionization spectroscopy (HRESIMS) data, NMR and electron-capture dissociation (ECD), respectively [18].

**Figure 2:**
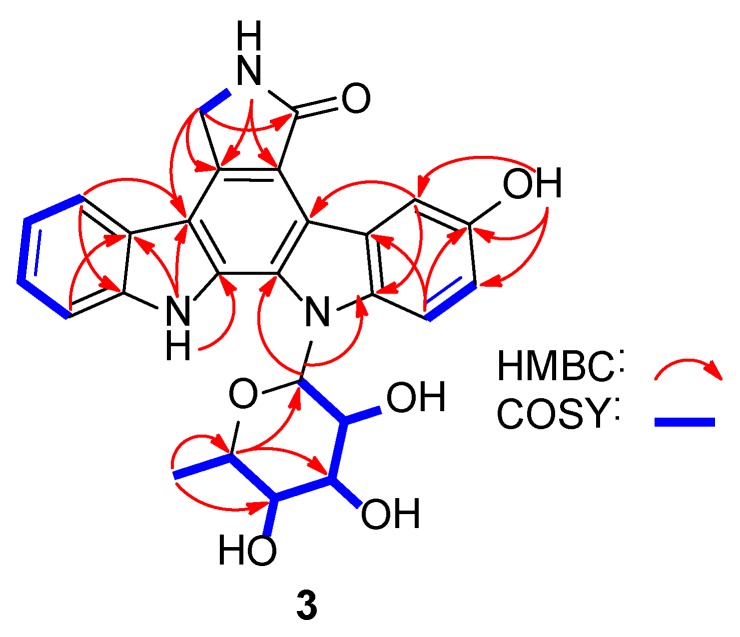
Key 2D NMR correlations for the structural assignment of **3** (blue: ^1^H-^1^H COSY connections, red: HMBC correlations).

3-Hydroxy-K252d (**3**) was isolated as a yellow amorphous powder. Its molecular formula was C_26_H_23_N_3_O_6_ based on the HRESIMS data at *m/z* 474.1657 [M + H]^+^ (Figure S7). Comparison of its NMR spectra with those of known ICZ analogues indicated that the ^1^H and ^13^C NMR data of **3** (Figures S2 and S3) were similar to K-252d [19]. The signal of the H-3 in K-252d was replaced by an exchangeable proton signal (*δ*_H_ 9.05), and *δ*_C-3_ increased to 150.8, leading to the assignment of C-3 bearing a hydroxy group. The structure of **3** was thus determined as 3-hydroxy K-252d, which was further confirmed by the 2D NMR correlations (Figure 2, Figures S4–S6). The configuration of a sugar moiety was determined to be l-rhamnose by GC-MS analysis of the acidic hydrolysates (Figure S1).

### 2.2. The Bioactivities of Compounds **1** and **3** from Streptomyces sp. OUCMDZ-3118

**Figure 3:**
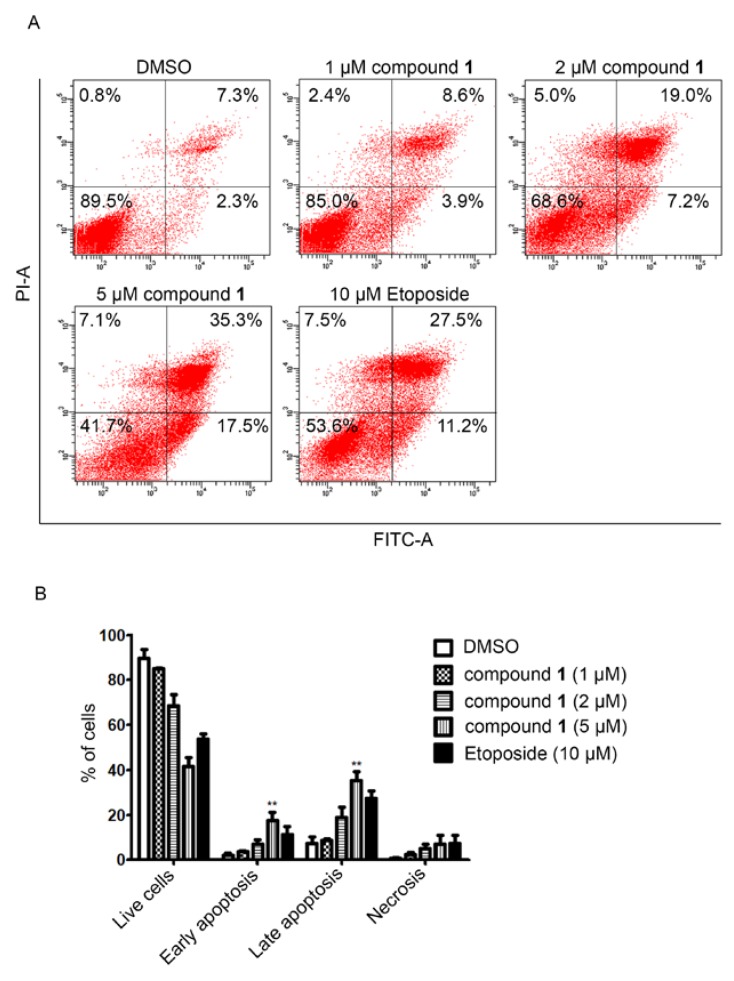
Compound **1** induced apoptosis in AGS cells. (**A**) Cells were treated with the indicated concentration of compound **1** and etoposide for 48 h. After being costained with annexin V and propidium iodine, cells were subjected to fluorescence activating cell sorting on a BD FACS Canto machine. (**B**) Percentage of cell population in each condition (double asterisk indicates statistical significance at ** *p* < 0.01).

**Figure 4:**
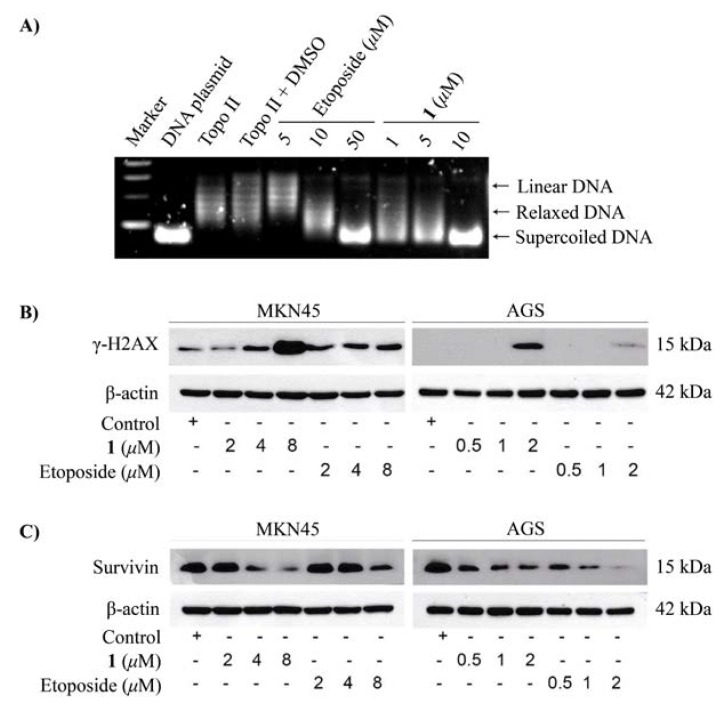
The effects of compound **1** on gastric cancer cell lines, AGS and MKN45. (**A**) The inhibitory effects of compound **1** and etoposide on topoisomerase IIα enzyme activity. (**B**) DNA damage (γ-H2AX) induced by compound **1** and etoposide. (**C**) The effect of compound **1** and etoposide on survivin expression.

The cytotoxic activity of compounds **1** and **3** were assayed against A549, MCF-7, and K562 tumor cells. Compound **1** was effective against the A549, K562, and MCF-7 cell lines with IC_50_ values of 0.51 ± 0.05 μM, 5.0 ± 0.2 μM, and 7.2 ± 0.6 μM, respectively, while compound **3** was moderately effective against the A549 and MCF-7 cell lines with IC_50_ values of 1.2 ± 0.05 μM and 1.6 ± 0.09 μM, respectively (Table 1). The positive control adriamycin (IC_50_), was tested against the A549, K562, and MCF-7 cell lines with IC_50_ values of 0.15 ± 0.03, 0.26 ± 0.07, and 0.21 ± 0.06 μM, respectively. We also assayed the cytotoxic effects and the molecular mechanism of compound **1** on two gastric cancer cell lines, AGS and MKN45. As a result, compound **1** exhibited potent cytotoxic activity than that of the clinically used etoposide at 48 h with the IC_50_ values of 1.7 ± 0.2 μM and 4.3 ± 1.0 μM in AGS and MKN45 cells, respectively. Whereas, the IC_50_ values at 48 h of etoposide in AGS and MKN45 cells were 8.6 ± 2.4 μM and >20 μM, respectively (Table 2). Treatment with compound **1** markedly induced apoptotic cell death in AGS cells (Figure 3). Compound **1** potently inhibited the activity on topoisomerase IIα enzyme (Figure 4A) leading to DNA damage as demonstrated by the accumulation of DNA damage marker, γ-H2AX (Figure 4B). In addition, compound **1** also reduced the expression of antiapoptotic protein survivin in two gastric cancer cells (AGS and MKN45) (Figure 4C). Taken together, these results suggest that compound **1** induced apoptotic cell death by inhibiting topoisomerase IIα enzyme activity-mediated DNA damage and reducing the expression of the antiapoptosis protein. Thus, compound **1** has the potential to be developed as a novel topoisomerase IIα inhibitor for anticancer therapy for the treatment of gastric cancer and other highly etoposide-resistant cancer cells. 

## 3. Experimental Section

### 3.1. General Experimental Procedures

Optical rotations were recorded with a JASCO P-1020 digital polarimeter (JASCO Corporation, Tokyo, Japan). UV spectra were recorded on a Beckman DU 640 (Beckman Coulter, Inc., Brea, CA, USA) spectrophotometer. ECD spectra were measured on JASCO J-715 spectrometer (JASCO Corporation, Tokyo, Japan). IR spectra were obtained on a Nicolet Nexus 470 (Thermo Nicolet Corporation, Madison, USA) spectrophotometer in KBr discs. NMR spectra were recorded on a JEOL JNM-ECP 600 (JEOL, Tokyo, Japan) or a Bruker Avance 500spectrometer (Bruker, Fallanden, Switzerland), and chemical shifts were referenced to the corresponding residual solvent signal (*δ*_H/C_ 2.50/39.52 for DMSO-*d*_6_). ESIMS recorded on a Q-TOF Ultima Global GAA076 LC mass spectrometer (Waters Asia, Ltd., Singapore). Semipreparative HPLC was performed using an ODS column [YMC-pak ODS-A, 10 × 250 mm, 5 μm, 4 mL/min]. TLC and column chromatography (CC) were performed on plates precoated with silica gel GF_254_ (10–40 μm) and over silica gel (200–300 mesh, Qingdao Marine Chemical Factory, Qingdao, China), and Sephadex LH-20 (Amersham Biosciences, Uppsala, Sweden), respectively. Vacuum liquid chromatography (VLC) was carried out over silica gel H (Qingdao Marine Chemical Factory). RPMI 1640 medium and antibiotic-antimycotic medium were purchased from Invitrogen (Carlsbad, CA, USA). Fetal bovine serum, RIPA buffer, proteinase inhibitor, and Super Signal West Pico chemi-luminescent substrate were purchased from Thermoscientific (Cramlington, UK). MTT (3-(4,5-dimethylthiazol-2-yl)-2,5-diphenyl-tetrazolium bromide) and etoposide were obtained from Sigma-Aldrich Chemical Co. (St. Louis, MO, USA). The Annexin V FITC apoptosis kit was purchased from BD bioscience (San Jose, CA, USA). Anti-γ-H2AX and antisurvivin antibodies were obtained from Cell Signaling Technology, Inc. (Danvers, MA, USA). Human topoisomerase IIα enzyme was purchased from TopoGen, Inc. (Port Orange, FL, USA). All other chemicals, unless otherwise stated, were purchased from Sigma-Aldrich Chemical Co. (St. Louis, MO, USA).

### 3.2. Strain OUCMDZ-3118 Material

The actinobacterial strain *Streptomyces* sp. OUCMDZ-3118 was isolated from a piece of deep-sea sediment (2061 m) collected in the South China Sea. The marine sediments (2 g) were air-dried for 15 days. The sediments were diluted with sterile water to a concentration of 10^−3^ g/mL, 100 μL, which was transformed on Gause’s synthetic agar media and cultured at 28 °C for 10 days. Then, a single colony was transferred to Gause’s synthetic agar media. It was identified according to its morphological characteristics and 16S rRNA gene sequences (GenBank access No. MG706259). Strain OUCMDZ-3118 was maintained at −80 °C in our laboratory.

### 3.3. Cultivation and Extraction of OUCMDZ-3118

The strain OUCMDZ-3118 was cultured in 100 × 500 mL Fernbach flasks each containing 150 mL of culture medium (2 g soluble starch , 5 g 5-hydroxy-l-tryptophan, 5 g yeast extract, 15 g soybean meal, 4 g NaCl, and 4 g CaCO_3_ dissolved in 1 L of sea water) and shaken at 180 rpm at 28 °C. Seven days after cultivation the broth was extracted three times with equal volumes of ethyl acetate (EtOAc). The EtOAc extract was concentrated in vacuo to give a dark brown gum (20.0 g).

### 3.4. Purification

The EtOAc extract (20.0 g) was separated into 9 fractions (Fr.1–Fr.9) on a silica gel VLC column, eluting with petroleum ether–CH_2_Cl_2_ (1:1, 0:1) and then with CH_2_Cl_2_–MeOH (100:1, 50:1, 30:1, 20:1, 10:1, 5:1, 1:1). Fraction 7 (890 mg) was purified by Sephadex LH-20 to afford three subfractions (Fr.7.1–Fr.7.3), eluting with CH_2_Cl_2_–MeOH (1:1). Fr.7.1 (41 mg) was purified over a ODS column (YMC-pack ODS-A, 10 × 250 mm, 5 μm, 4 mL/min) using the solvent system of 65% MeCN containing 1.5‰ trifluoroacetic acid (TFA) to yield compound **3** (3.0 mg, *t*_R_ = 12 min). Fraction 8 (435 mg) was separated on Sephadex LH-20 with CH_2_Cl_2_–MeOH (1:1) into four subfractions (Fr.8.1–Fr.8.4). Fr.8.1 (195 mg) was further purified by HPLC on a ODS column (YMC-pack ODS-A, 10 × 250 mm, 5 μm, 4 mL/min) using the solvent system of 70% MeOH to afford compounds **1** (25 mg, *t*_R_ = 5.6 min) and **2** (1.2 mg, *t*_R_ = 5.8 min).

*3-Hydroxyholyrine A* (**1**): yellow amorphous powder; ${\left[\alpha \right]}_{D}^{25}$ –32.8 (*c* 0.2, MeOH); UV (MeOH) λ_max_ (log *ε*) 228 (3.04), 295 (4.05), 342 (1.33) and 375 (0.98) nm; ECD (0.0022*M*, MeOH) λ_max_ (Δ*ε*) 209 (+8.43), 227 (−4.21), 263 (+6.54), 286 (−5.52), 295 (−1.49), 304 (−6.34) and 371 (+3.86) nm; IR (KBr) *ν*_max_ 3421, 1679, 1649, 1458, 1205, 1136 cm^−1^; ^1^H (600 MHz, DMSO-*d*_6_): *δ* 7.73 (1H, d, *J* = 8.8 Hz, H-1), 7.01 (1H, dd, *J* = 8.8, 2.4 Hz, H-2), 8.87 (1H, d, *J* = 2.4 Hz, H-4), 8.52 (1H, s, NH-6), 4.97 (2H, s, H-7), 8.05 (1H, d, *J* = 7.8 Hz, H-8), 7.31 (1H, ‘t’ like, *J* = 7.8, 6.9 Hz, H-9), 7.49 (1H, ‘t’ like, *J* = 8.2, 6.9 Hz, H-10), 7.72 (1H, d, *J* = 8.2 Hz, H-11), 11.69 (1H, s, NH-12), 6.57 (1H, dd, *J* = 11.0, 3.0 Hz, H-1′), 1.97 (1H, m, H-2′), 2.45 (1H, m, H-2′), 4.11 (1H, m, H-3′), 4.08 (1H, brs, H-4′), 4.60 (1H, ‘q’ like, *J* = 7.0 Hz, H-5′), 1.58 (1H, d, *J* = 7.0 Hz, H-6′), 9.19 (1H, s, HO-3), 8.17 (2H, brs, NH_2_-3′), 7.60 (1H, brs, HO-4′); ^13^C (150 MHz, DMSO-*d*_6_): *δ* 110.0 (CH, C-1), 115.2 (CH, C-2), 151.3 (C, C-3), 110.5 (CH, C-4), 123.1 (C, C-4a), 117.4 (C, C-4b), 118.7 (C, C-4c), 172.4 (C, C-5), 45.9 (CH_2_, C-7), 133.9 (C, C-7a), 117.4 (C, C-7b), 121.9 (C, C-7c), 121.3 (CH, C-8), 120.1 (CH, C-9), 125.2 (CH, C-10), 111.9 (CH, C-11), 139.2 (C, C-11a), 127.4 (CH, C-12a), 124.6 (C, C-12b), 132.9 (C, C-13a), 75.3 (CH, C-1′), 30.8 (CH_2_, C-2′), 45.9 (CH, C-3′), 66.2 (CH, C-4′), 76.5 (CH, C-5′), 14.1 (CH_3_, C-6′); HRESIMS *m*/*z* 457.1875 [M + H]^+^ (calcd for C_26_H_25_N_4_O_4_, 457.1870).

*3′-N-acetyl-3-hydroxyholyrine A* (**2**): yellow amorphous powder; ${\left[\alpha \right]}_{D}^{25}$ –25.8 (*c* 0.1, MeOH); UV (MeOH) λ_max_ (log *ε*) 226 (2.31), 296 (2.99), 342 (0.95) and 376 (0.77) nm; ECD (0.0020*M*, MeOH) λ_max_ (Δ*ε*) 209 (+6.78), 227 (–3.66), 263 (+5.36), 283 (–4.86), 295 (–2.95), 306 (–5.55) and 370 (+3.66) nm; IR (KBr) *ν*_max_ 3359, 1682, 1459, 1392, 1206, 1139 cm^−1^; ^1^H (600 MHz, DMSO-*d*_6_): *δ* 7.77 (1H, d, *J* = 8.8 Hz, H-1), 6.97 (1H, dd, *J* = 8.8, 2.5 Hz, H-2), 8.84 (1H, d, *J* = 2.5 Hz, H-4), 8.45 (1H, s, NH-6), 4.96 (2H, s, H-7), 8.03 (1H, d, *J* = 7.8 Hz, H-8), 7.31 (1H, ‘t’ like, *J* = 7.8, 7.0 Hz, H-9), 7.48 (1H, ‘t’ like, *J* = 8.0, 7.0 Hz, H-10), 7.67 (1H, d, *J* = 8.0 Hz, H-11), 12.01 (1H, s, NH-12), 6.58 (1H, dd, *J* = 11.2, 3.4 Hz, H-1′), 1.74 (1H, m, H-2′), 2.46 (1H, m, H-2′), 4.64 (1H, m, H-3′), 3.90 (1H, brs, H-4′), 4.49 (1H, ‘q’ like, *J* = 7.2 Hz, H-5′), 1.58 (1H, d, *J* = 7.2 Hz, H-6′), 9.10 (1H, s, OH-3), 7.89 (1H, brs, NH-3′), 6.91 (1H, brs, OH-4′). 1.81 (3H, s, H-2′’); ^13^C (150 MHz, DMSO-*d*_6_): *δ* 110.1 (CH, C-1), 115.2 (CH, C-2), 151.0 (C, C-3), 110.3 (CH, C-4), 123.0 (C, C-4a), 117.2 (C, C-4b), 118.5 (C, C-4c), 172.5 (C, C-5), 45.3 (CH_2_, C-7), 133.7 (C, C-7a), 117.4 (C, C-7b), 121.9 (C, C-7c), 121.3 (CH, C-8), 120.1 (CH, C-9), 125.2 (CH, C-10), 111.9 (CH, C-11), 139.3 (C, C-11a), 127.6 (CH, C-12a), 124.8 (C, C-12b), 133.1 (C, C-13a), 76.1 (CH, C-1′), 32.2 (CH_2_, C-2′), 45.3 (CH, C-3′), 68.2 (CH, C-4′), 76.7 (CH, C-5′), 14.6 (CH_3_, C-6′), 18.1 (CH_3_, C-2′’); HRESIMS *m*/*z* 499.1970 [M + H]^+^ (calcd for C_28_H_27_N_4_O_5_, 499.1976).

*3-Hydroxy-K252d* (**3**): yellow amorphous powder; ${\left[\alpha \right]}_{D}^{25}$ +28.0 (*c* 0.2, MeOH); UV (MeOH) λ_max_ (log *ε*) 226 (2.99), 296 (3.93), 341 (1.32) and 376 (0.97) nm; IR (KBr) *ν*_max_ 3428, 1681, 1457, 1210, 1125 cm^−1^; ^1^H (600 MHz, DMSO-*d*_6_): *δ* 7.48 (1H, d, *J* = 8.8 Hz, H-1), 6.98 (1H, dd, *J* = 8.8, 2.5 Hz, H-2), 8.85 (1H, d, *J* = 2.3 Hz, H-4), 8.45 (1H, s, NH-6), 4.95 (2H, s, H-7), 8.03 (1H, d, *J* = 7.8 Hz, H-8), 7.29 (1H, ‘t’ like, *J* = 7.8, 7.0 Hz, H-9), 7.46 (1H, ‘t’ like, *J* = 8.1, 7.0 Hz, H-10), 7.57 (1H, d, *J* = 8.0 Hz, H-11), 11.59 (1H, s, NH-12), 6.26 (1H, dd, *J* = 9.5 Hz, H-1′), 4.43 (1H, dd, *J* = 9.5, 3.1 Hz, H-2′), 4.15 (1H, ddd, *J* = 3.2, 3.2, 3.1 Hz, H-3′), 4.02 (1H, brs, H-4′), 4.44 (1H, dq, *J* = 3.3, 7.3 Hz, H-5′), 1.66 (1H, d, *J* = 7.3 Hz, H-6′), 9.05 (1H, s, HO-3), 5.40 (1H, d, *J* = 3.2 Hz, HO-3′), 6.72 (1H, brs, HO-4′), 6.91 (1H, brs, HO-4′). 1.81 (3H, s, H-2″); ^13^C (150 MHz, DMSO-*d*_6_): *δ* 110.2 (CH, C-1), 115.1 (CH, C-2), 150.8 (C, C-3), 110.2 (CH, C-4), 122.9 (C, C-4a), 117.4 (C, C-4b), 118.7 (C, C-4c), 172.5 (C, C-5), 45.3 (CH_2_, C-7), 133.5 (C, C-7a), 114.7 (C, C-7b), 122.1 (C, C-7c), 121.2 (CH, C-8), 120.0 (CH, C-9), 125.2 (CH, C-10), 111.3 (CH, C-11), 139.1 (C, C-11a), 127.8 (CH, C-12a), 125.2 (C, C-12b), 134.9 (C, C-13a), 77.4 (CH, C-1′), 67.2 (CH, C-2′), 71.9 (CH, C-3′), 71.6 (CH, C-4′), 76.6 (CH, C-5′), 15.5 (CH_3_, C-6′). ^1^H and ^13^C NMR data, see Table S1; HRESIMS *m*/*z* 474.1657 [M + H]^+^ (calcd for C_26_H_24_N_3_O_6_, 474.1660).

### 3.5. Sugar Analysis for **3**

HCl (1*M*, 2.0 mL) was added to a solution of 3-hydroxy-K252d (**3**, 1.0 mg) in ethanol (0.3 mL) and benzene (3 mL) at room temperature. The reaction mixture was stirred at 50 °C for 2 h. Then, it was diluted with H_2_O (3 mL) and extracted with EtOAc (6 mL × 3). The aqueous layer was dried in vacuo after neutralization with 2 N NaOH and chromatographed over Sephadex LH-20 eluted with MeOH to desalt. The hydrolysate and l-cysteine methyl ester (1.0 mg) were dissolved in anhydrous pyridine (1 mL) and the resulting mixture was stirred at 60 °C for 1 h. A 3:1 mixture of HMDS-TMCS (100 µL) was added, and the solution was stirred for 30 min. Hexanes (1.5 mL) and water (1 mL) were added to the solution. The hexane layer was dried over anhydrous Na_2_SO_4_ and subjected to GC-MS analysis (30 mm × 0.32 mm × 0.25 µm HP-5 MS column: He, 1 mL/min; 40 °C for 2 min, 40–250 °C, ∆ 15 °C/min, 250 °C for 10 min), which permitted the identification of the l-rhamnose derivative (*t*_R_ = 18.10 min) by comparison with standard l-rhamnose derivative (*t*_R_ = 18.11 min).

### 3.6. Supercoiled Plasmid DNA Relaxation Assay

The activity of human DNA topoisomerase IIα was determined as previously described [20]. Supercoiled pBR322 plasmids were incubated with various concentrations of compound **1** and two units of human topoisomerase IIα enzyme in cleavage buffer (0.5 M tris-HCL (pH 8.0), 1.5 M NaCl, 0.1 M MgCl_2_, 5 mM dithiothreitol, 300 μg/mL BSA, and 20 mM ATP in water) at 37 °C for 1 h. The reaction was then terminated by adding 2 μL of 10% SDS and 1 μL of protein kinase 250 mg/mL. The samples were electrophoresed using 1% agarose gel, stained with SYBR safe dye, and photographed under gel documentation machine.

### 3.7. Apoptosis Analysis

Cell apoptosis was analyzed using an Annexin V-FITC Apoptosis Detection Kit and fluorescence activated cell sorting analysis. Briefly, AGS cells were cultured in six-well plates for 24 h before treatment with various concentrations of compound **1** and 10 μM of etoposide. After 48 h, cells were trypsinized and washed with ice-cold phosphate buffer saline (PBS). Cells were co-stained with propidium iodide and annexin V for 15 min at room temperature in the dark and then were analyzed by BD FACSCanto flow cytometry.

### 3.8. Western Blotting Analysis

MKN45 and AGS cells were cultured in six-well plates for 24 h and treated with various concentrations of compound **1** and etoposide for 24 h. Cells were then lysed with lysis buffer and the concentrations of the proteins were measured using the bicinchoninic acid (BCA) assay. The equal amount of protein was separated on 10% SDS-polyacrylamide gel electrophoresis (SDS-PAGE) and transferred into a polyvinylidene difluoride (PVDF) membrane. The non-specific protein binding was blocked by incubating membranes with 5% blotting grade non-fat dry milk for 1 h at room temperature. After washing with TBST, the membranes were incubated with desired primary antibodies overnight at 4 °C, and washed with TBST for 5 times, before incubation with a HRP-conjugated secondary antibody at room temperature for 1 h. The signals were detected using Luminata Crescendo Western HRP Substrate (Millipore, Billerica, MA, USA). Anti-β-actin was used as a loading control.

## 4. Conclusions

In summary, we utilized precursor-directed biosynthesis to obtain a new and bioactive ICZ **3**. Our study is considered as one of the powerful advanced approaches to obtain diverse natural products with a variety of biological and therapeutic activities. Biological and mechanic studies revealed compound **1** to be a new topoisomerase IIα inhibitor for the treatments of gastric cancer and other highly etoposide-resistant cancers. 

## Figures and Tables

**Table 1:** IC_50_ (μM) of **1** and **3** against A549, K562, and MCF-7 cell lines.

Compound	1	3
A549	0.51 ± 0.05	1.2 ± 0.05
K562	5.0 ± 0.2	>10
MCF-7	7.2 ± 0.6	1.6 ± 0.09

**Table 2:** IC_50_ values of **1** and etoposide against MKN45 and AGS cell lines.

Cell Line	Incubation Time (hours)	IC_50_ (μM)	
		Etoposide	1
MKN45	24	>20	14.2 ± 1.8
	48	>20	4.3 ± 1.0
	72	16.0 ± 2.3	4.7 ± 0.4
AGS	24	>20	4.9 ± 1.3
	48	8.6 ± 2.4	1.7 ± 0.2
	72	2.9 ± 0.5	2.1 ± 0.3

## Supplementary Materials

The following are available online at http://www.mdpi.com/1660-3397/16/5/168/s1. Bioassay protocols, Figure S1: ^1^H-NMR spectrum of 3-hydroxy-K252d (**3**) in DMSO-*d*_6_, Figure S2: ^1^H-NMR spectrum of 3-hydroxy-K252d (**3**) in DMSO-*d*_6_, Figure S3: ^13^C-DEPTQ-NMR spectrum of 3-hydroxy-K252d (**3**) in DMSO-*d*_6_, Figure S4: HSQC spectrum of 3-hydroxy-K252d (**3**) in DMSO-*d*_6_, Figure S5: ^1^H-^1^H COSY spectrum of 3-hydroxy-K252d (**3**) in DMSO-*d*_6_, Figure S6: HMBC spectrum of 3-hydroxy-K252d (**3**) in DMSO-*d*_6_, Figure S7: HR-ESIMS of 3-hydroxy-K252d (**3**).
