# Supplementary material for: Precursor-Directed Generation of Indolocarbazoles with Topoisomerase IIα Inhibitory Activity
Source: Mar Drugs. 2018 May 17;16(5):168. doi: 10.3390/md16050168 (PMC5983299; doi:10.3390/md16050168)

Precursor-Directed Generation of Indolocarbazoles with Topoisomerase IIα Inhibition

Cong Wang [1,2,†], Adeep Monger [3,†], Liping Wang [4], Peng Fu [1], Pawinee Piyachaturawat [3], Arthit Chairoungdua [3,*] and Weiming Zhu [1,6,*]

[1] Key Laboratory of Marine Drugs, Ministry of Education of China, School of Medicine and Pharmacy, Ocean University of China, Qingdao 266003, China; wangcong123206@163.com (C.W.); fupeng@ouc.edu.cn (P.F.)

[2] Guangxi Key Laboratory of Chemistry and Engineering of Forest Products, School of Chemistry and Chemical Engineering, Guangxi University for Nationalities, Nanning 530006, China

[3] Toxicology Graduate Program, Department of Physiology, Faculty of Science, Mahidol University, Bangkok 10400, Thailand; adeepmonger11@gmail.com (A.M.); pawinee.pia@mahidol.ac.th (P.P.)

[4] Key Laboratory of Chemistry for Natural Products of Guizhou Province and Chinese Academy of Sciences, Guiyang 550002, China; lipingw2006@163.com

[5] Toxicology Graduate Program, Excellent Center for Drug Discovery (ECDD), Faculty of Science, Mahidol University, Bangkok 10400, Thailand

[6] Laboratory for Marine Drugs and Bioproducts, Qingdao National Laboratory for Marine Science and Technology, Qingdao 266003, China

[*] Correspondence: arthit.chi@mahidol.ac.th (A.C.); weimingzhu@ouc.edu.cn (W.Z.); Tel./Fax: +86-532-82031268 (W.Z.)

[†] These authors contributed equally to this paper.

Table of contents

Bioassay Protocols……S2

Figure S1. GC-MS diagram of the directives of standard L-rhamnose and the sugar from compound **3**……S3

Figure S2. ^{1}H-NMR spectrum of 3-hydroxy-K252d (**3**) in DMSO-d_6……S4

Figure S3. ^{13}C-DEPTQ-NMR spectrum of 3-hydroxy-K252d (**3**) in DMSO-d_6……S5

Figure S4. HSQC spectrum of 3-hydroxy-K252d (**3**) in DMSO-d_6……S6

Figure S5. ^{1}H-^{1}H COSY spectrum of 3-hydroxy-K252d (**3**) in DMSO-d_6……S7

Figure S6. HMBC spectrum of 3-hydroxy-K252d (**3**) in DMSO-d_6……S8

Figure S7. HR-ESIMS of 3-hydroxy-K252d (**3**)……S9

Bioassay Protocols

Cell Culture. Human gastric cancer cell lines, MKN45 and AGS cells were purchased from Japanese cell bank (JCRB, Japan) and from American Type culture collection (ATCC, Manassas, VA), respectively. They were cultured in RPMI medium supplemented with 10% fetal bovine serum and 1% antibiotic (100 U/ml of penicillin and 100 μg/mL of streptomycin) in a humidified 5% CO_2 incubator (Invitrogen, Carlsbad, CA) at 37 °C.

Cell Viability Assay. The effect of compound on cell viability was determined by using the colorimetric MTT assay. MKN45 and AGS cells were seeded in a 96-well plate at a concentration of 2×10^4 cells/well and 7×10^3 cells/well, respectively. Cells were cultured for 24 h and then treated with various concentrations of compounds ranging from 0.1−20 μM at different time courses (24, 48 and 72 h). In the control group, cells were cultured in medium containing equivalent amount of DMSO required to dissolve the compounds (less than 0.05%). At the end of incubation, medium was removed, cells were incubated with 0.5 mg/mL of 3-(4, 5-dimethylthiazol-2-yl)-2,5-diphenyltetrazolium in a humidified 5% CO_2 incubator at 37 °C for 3 h. The dark blue formazan was dissolved with DMSO and measured at the wavelength of 540 nm using a Multiskan™ GO Microplate Spectrophotometer (Thermoscientific, Cramlington, UK). The result was calculated as % of cell viability.

Cytotoxic Assay. The cytotoxicity on the human tumor cells (MCF-7, A549 and K562) were assayed by MTT (3-[4,5-dimethyl thiazol-2-yl]-2,5-diphenyl tetrazolium bromide) and CCK-8 (Cell Counting Kit-8) methods.[S1] A549 and MCF-7 cell lines was grown in RPMI-1640 supplemented with 10% FBS under a humidified atmosphere of 5% CO_2 and 95% air at 37 ℃. Cell suspension, 100 μL, at a density of 3×10^4 cell mL^{-1} for tumor cells, was plated in 96-well microtiter plates and exposed to different concentrations of compounds in triplicate for 72 h. The initial concentrations of compounds were 2 mg/mL in DMSO, and then were diluted into 625, 125, 5, and 1 μg/mL with RPMI-1640 medium, respectively. The experiments were divided into blank control, testing compounds and the positive control groups, and each group was set up three parallels. Then, 10 μL of PBS containing MTT with the final concentration of 0.5 mg/mL was added to each well. After 4 h incubation at 37 ℃, the supernatant was removed and 150 μL of DMSO was added to each well to solubilize the formazan crystals. After vigorous shaking, absorbance values were measured in a microplate reader (Bio-Rad, USA) at 570 nm. In the CCK-8 assay, 100 μL of the K562 cell suspensions were plated in 96-well plates at a density of 3×10^4 cell mL^{-1}, respectively. Then, 100 μL of the test solutions (in DMSO) were added to each well and further incubated for 72 h. The CCK-8 solution (10 μL) was then added to each well and incubated for 6 h. Absorbance was then measured at 450 nm. The cytotoxicity was represented at these concentrations as $[(OD_{blank} - OD_{drug})/OD_{blank} \times 100\%]$. The half maximal cytotoxic concentration (CC_{50}) defined as the compound concentration necessary to induce cell cytotoxicity by 50%, was calculated by SPSS (Statistical Product and Service Solutions) v19.0 software.

GC-MS diagram of the directives of standard L-rhamnose and the sugar from compound 3

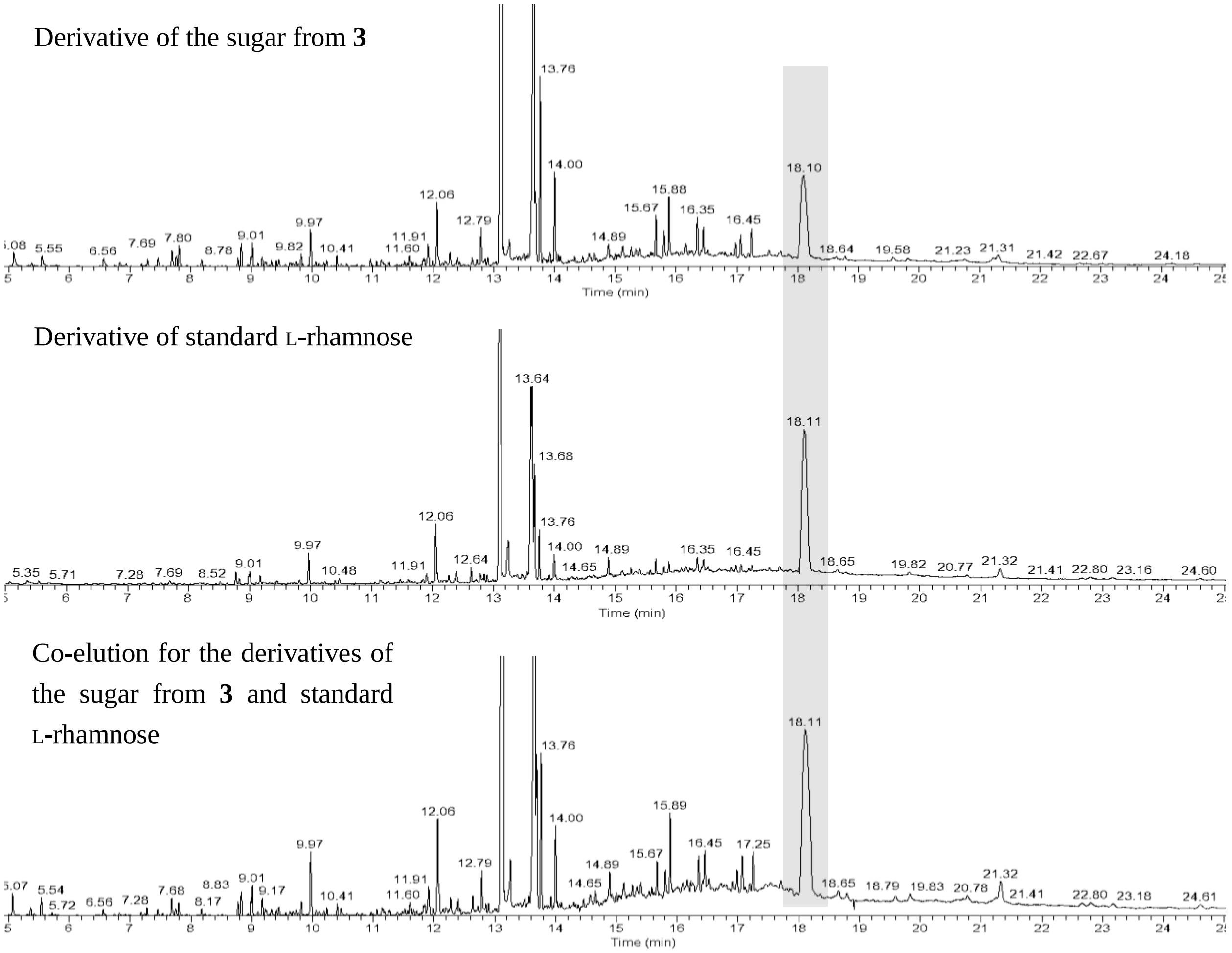

Figure S1. GC-MS diagram of the directives of standard L-rhamnose and the sugar from compound **3**.

Figure S2. ^{1}H-NMR spectrum of 3-Hydroxy-K252d (**3**) in DMSO-d_6

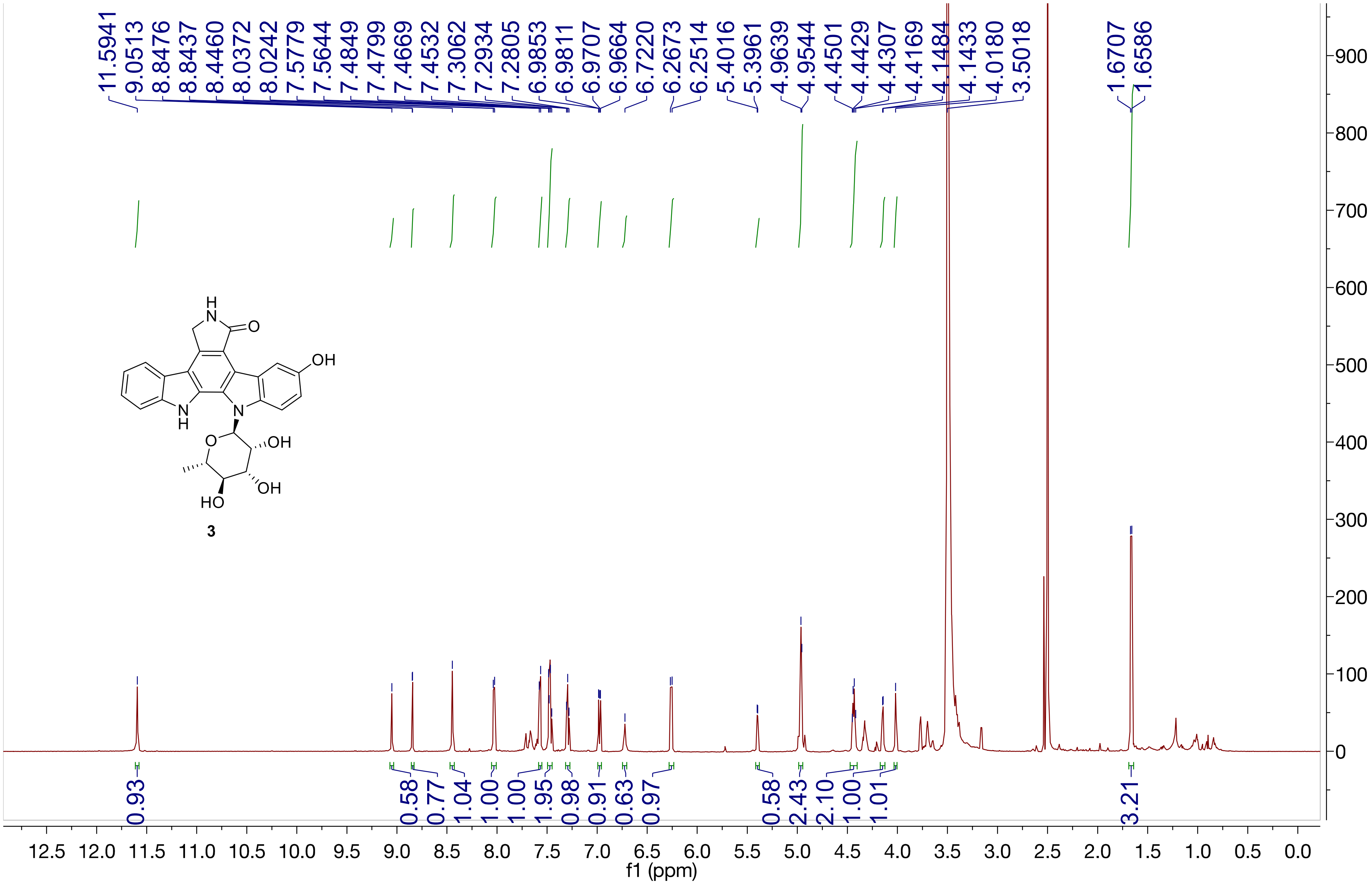

Figure S3. ^{13}C-DEPTQ-NMR spectrum of 3-Hydroxy-K252d (**3**) in DMSO-d_6

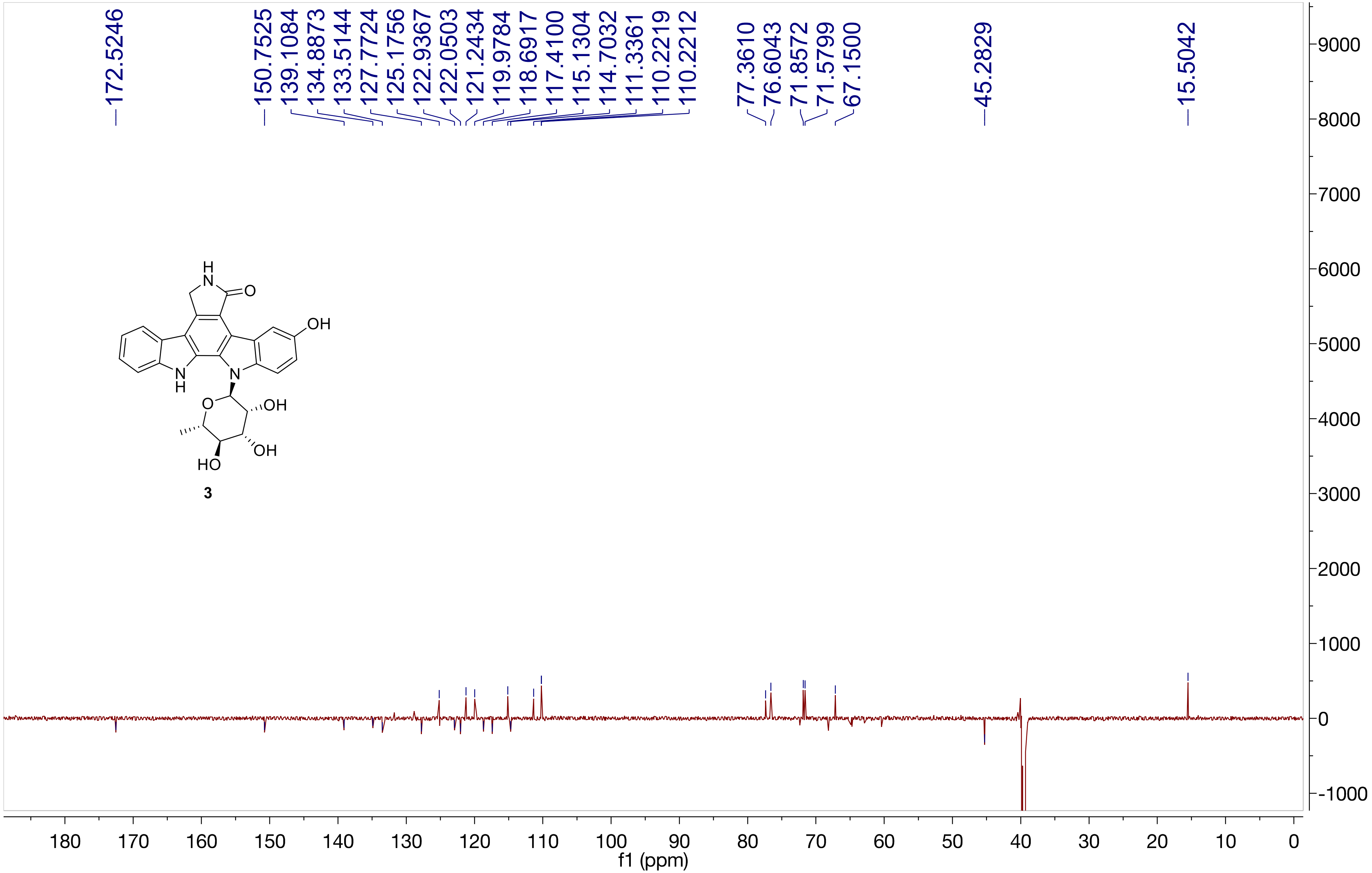

Figure S4. HSQC spectrum of 3-Hydroxy-K252d (**3**) in DMSO-d_6

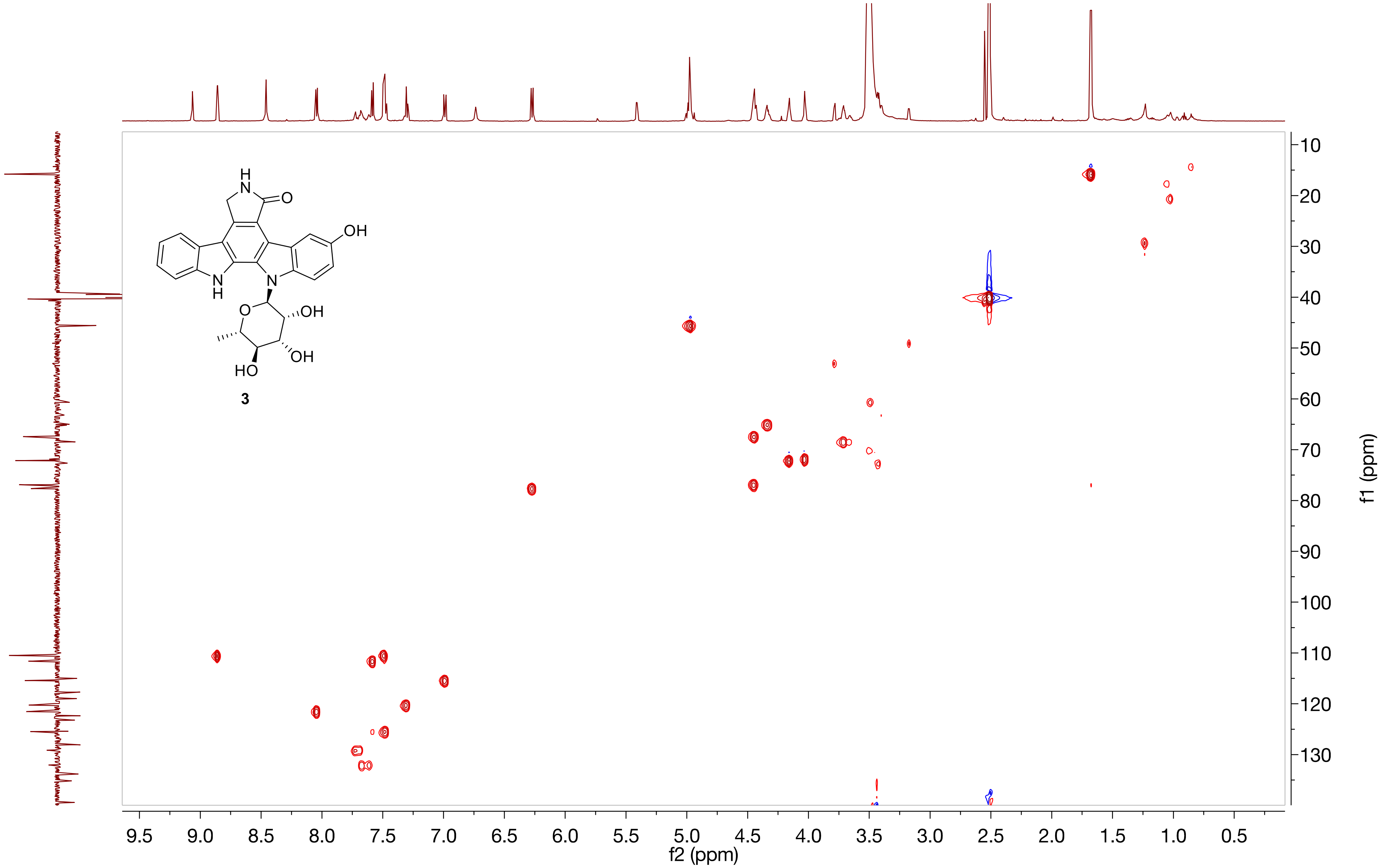

Figure S5. ^1H-^1H COSY spectrum of 3-Hydroxy-K252d (**3**) in DMSO-d_6

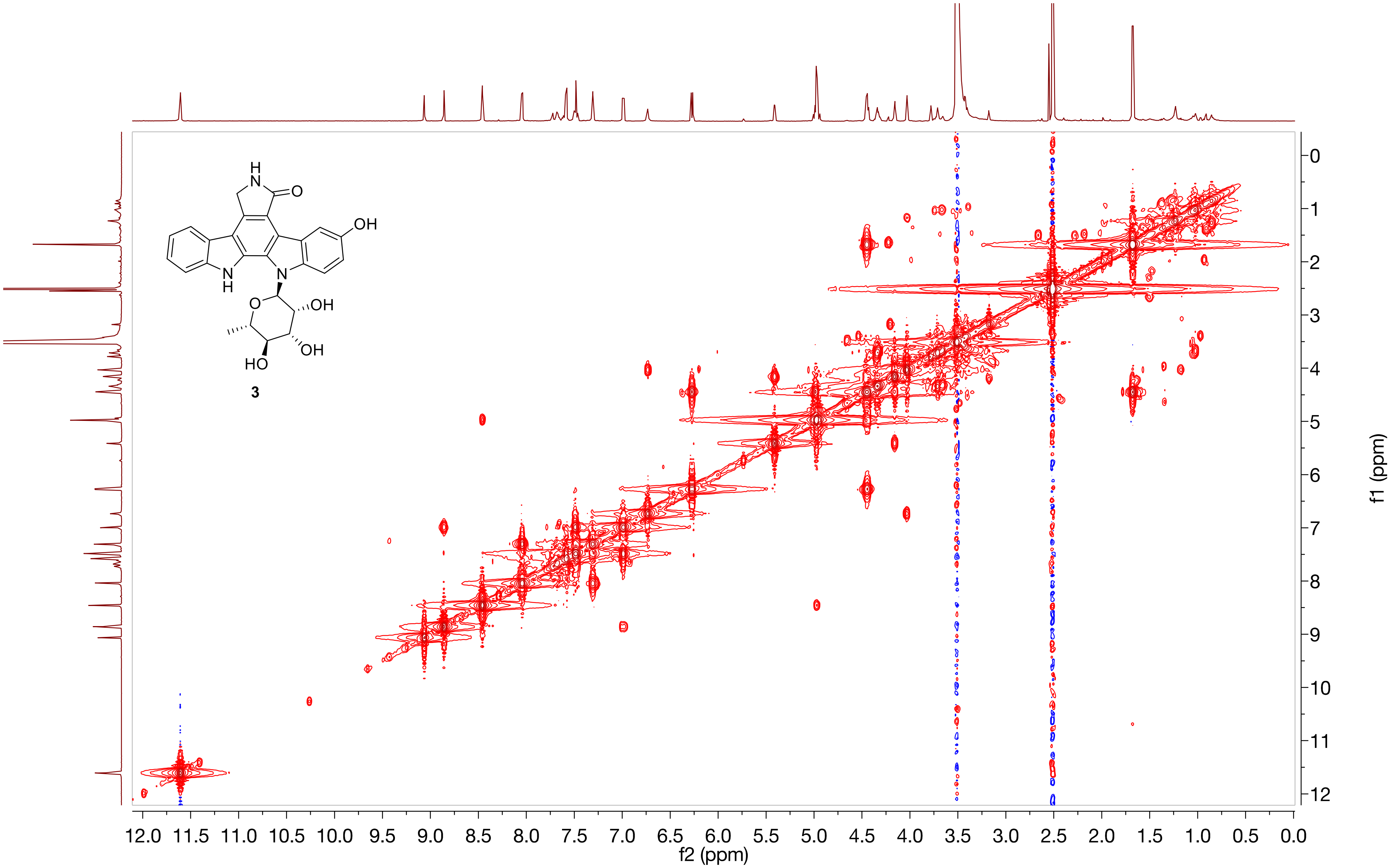

Figure S6. HMBC spectrum of 3-Hydroxy-K252d (**3**) in DMSO-*d*$_6$

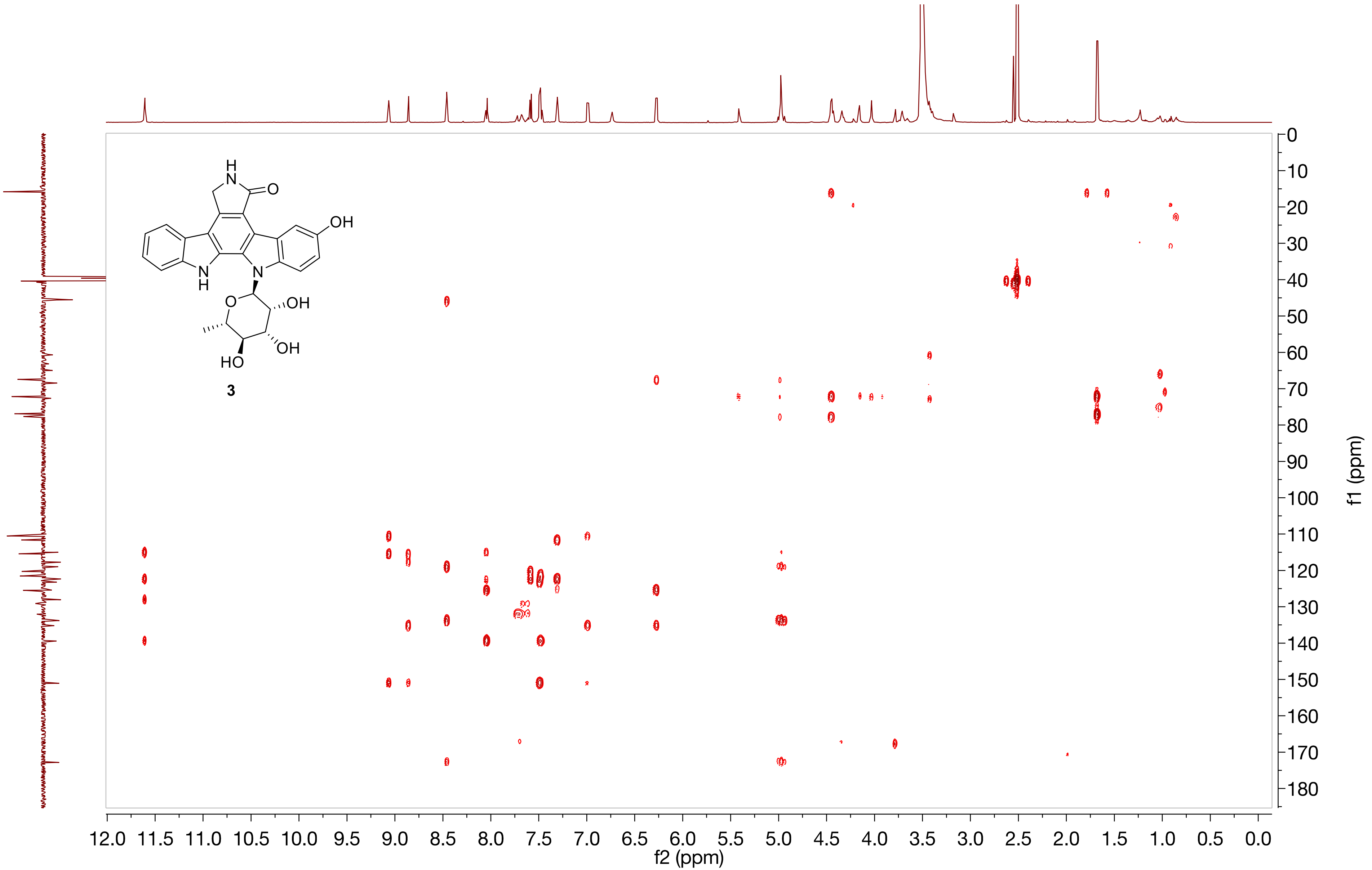

Figure S7. HR-ESIMS of 3-Hydroxy-K252d (**3**)

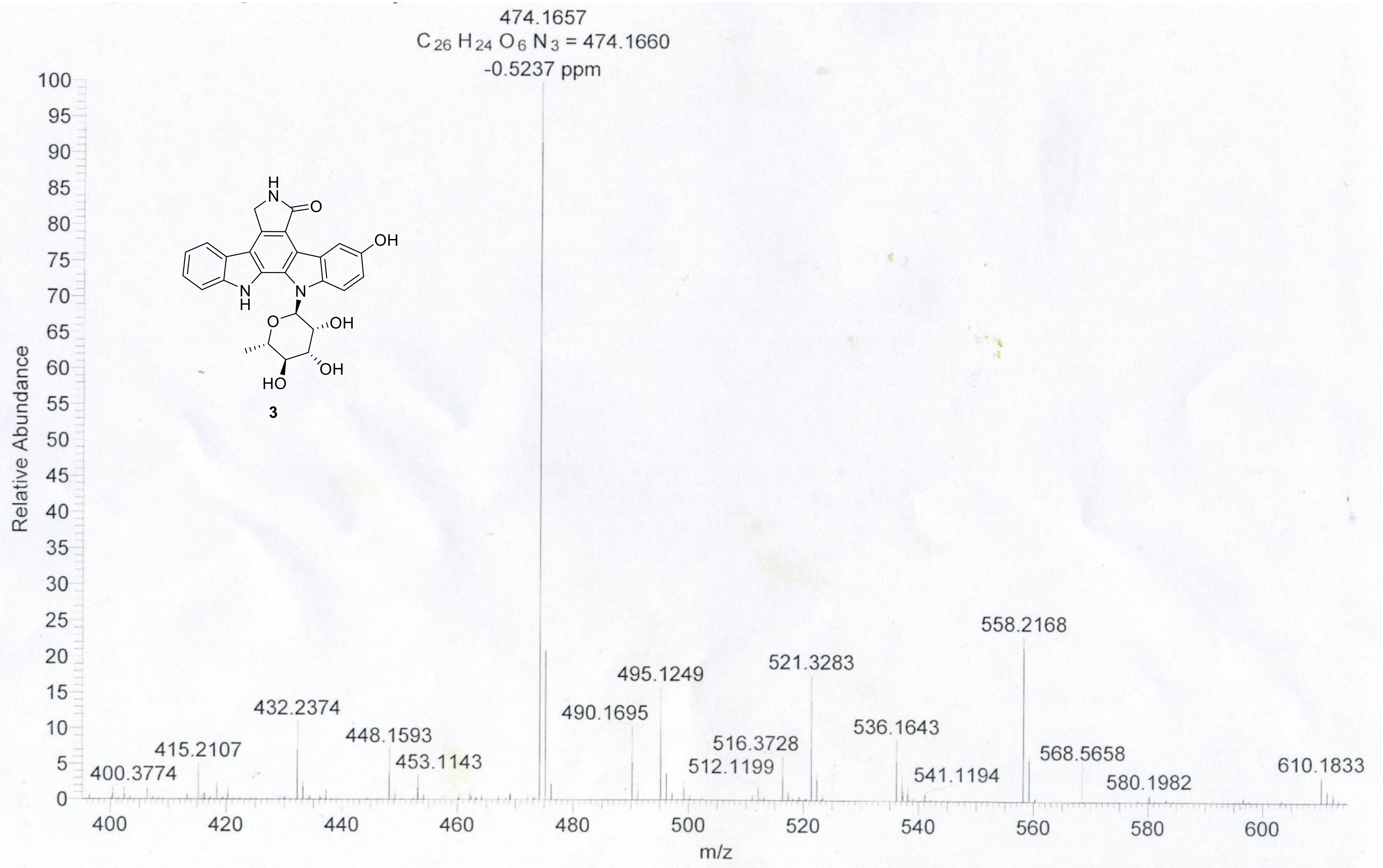

Supplement: Supplementary file 1 [file marinedrugs-16-00168-s001.pdf]
